
JOURNAL INFORMATION
==============================
NLM Title Abbreviation: Tex Med J (Austin)
NLM Journal ID: 21130590R

Texas Medical Journal

ISSN: 0892-8495
Publisher: Biomedical Journal Digitization Project

ARTICLE INFORMATION
==============================
PMCID: PMC9522052
Article version: 1
Subjects: Book Notices

A Practical Treatise on Nervous and Mental Diseases

Print publication date: 1897 Aug
Volume: 13
Issue: 2
First page: 101
Last page: 101
License: This work is free of known copyright restrictions. For more information, please see PMC Back Issue Digitization.
License URL: https://creativecommons.org/publicdomain/mark/1.0/

==============================

---

A Practical Treatise on Nervous and Mental Diseases. By Landon Carter Gray, M. D., Professor of Diseases of the Mind and Nervous System in the New York Polyclinic. New (2nd) Edition. In one octavo volume of 728 pages, with 172 engravings and 3 colored plates. Price, in cloth, $4.75; leather, $5.75. Publishers: Lea Brothers & Co., 706, 708 and 710 Sansom Street, Philadelphia.

The two subjects treated of in this volume, viz: Nervous diseases and mental diseases, although constituting two distinct specialties and usually treated of in separate volumes, are so intimately connected that they can very properly be considered in one volume, as they are done here; indeed many will be pleased with this arrangement of the two subjects. This edition will be found carefully revised and brought up to date, and will, no doubt, be found as satisfactory to the many friends of the distinguished author, as was the first edition. The whole field of diseases of the mind and of the nervous system is carefully gone over; the book being well written, terse and explicit, is an authority on the subjects discussed. The author's many years of study and teaching, and his long practical experience in the treatment of these diseases eminently qualifies him for the work of author of this volume. The book will be a valuable addition to any medical man's library, and the specialist on nervous or mental diseases cannot afford to be without it. S. E. H.

---

### Publishers' Notes.

**Rheumatisms and Neuralgias of Malarial Origin.**— "Whatever may be the first cause of the group of pathological conditions, which are usually classed under the head of malarial diseases, and in the treatment of which antipyretics play so important a part, there are certain sequelæ of malaria for which all antipyretics are powerless, from quinine down to the latest products of German dye works. Such are the rheumatisms and neuralgias accompanying or following attacks of malarial fever
